# Outcome of laparoscopic feeding jejunostomy, comparison of a pure laparoscopic technique with Witzel’s tunnel to open technique: a retrospective cohort study

**DOI:** 10.1186/s12893-024-02607-9

**Published:** 2024-10-14

**Authors:** Sumet Komek, Sirikan Limpakan, Peticha Tanprasert, Bandhuphat Chakrabandhu

**Affiliations:** Department of Surgery, Faculty of Medicine, Chiang Mai University, 110 Intawaroros Road, Sriphoom Sub-District, Muang District, Chiang Mai, 50200 Thailand

**Keywords:** Laparoscopic, Feeding jejunostomy, Enteric access

## Abstract

**Introduction:**

Obstructive upper GI cancer commonly uses feeding jejunostomy as a standard procedure. Surgeons implemented laparoscopic feeding jejunostomy via minimally invasive surgery, employing a variety of techniques. This study assessed the perioperative results, safety, and costs associated with laparoscopic versus open jejunostomy surgeries. We used only Witzel’s tunnel and standard laparoscopic instruments.

**Patients and methods:**

We collected data from all patients who underwent feeding jejunostomy between January 2016 and June 2018. We recorded pertinent data on baseline, surgical outcomes, postoperative results, complications, and costs. The study excluded patients with jejunostomy as a conversion or an addition.

**Result:**

We divided the 74 patients into 2 groups: 30 underwent laparoscopy and 44 underwent open surgery. The mean operational times were 89.67 and 91.64 min and showed no statistically significant difference (*p* = 0.678). The mean morphine dosage was significantly lower in the laparoscopic group (3.3 vs. 7.19, p = < 0.001). Laparoscopic surgery lowered the median time of feeding initiation, feeding accomplished, and postoperative stay, although none of these reached statistical significance. There were significantly higher surgical expenses in the laparoscopic group (16,410 vs. 11,685 Thai Baht) (*p* < 0.001); however, median overall expenditures did not significantly differ (105,147 vs. 116,198 Thai Baht) (*p* = 0.387). Laparoscopic versus open surgery had similar incidences of postoperative complications (20% vs. 25%, *p* = 0.846). The feeding tube catheter location was infection-free in all patients in our study.

**Conclusion:**

Laparoscopic jejunostomy feeding was safe, and postoperative morphine consumption was lower. Increasing operational costs did not have a significant impact on overall expenditures. Witzel’s tunnel may reduce jejunostomy site infections.

## Background

The high prevalence of malnutrition and cachexia in upper gastrointestinal (GI) cancer is a direct result of tumor obstructive symptoms [1]. Nutritional treatment is essential for affected patients, encompassing both parenteral and enteral feeding. Enteral nutrition offers numerous advantages over parenteral nourishment when it is feasible [2]. When a tumor disrupts the natural path for eating, an enteric access method is required, necessitating the creation of an artificial enteric feeding route [3]. 

In this group of patients, the procedure mostly relied on feeding jejunostomy. There are numerous benefits to this procedure when comparing it to the gastrostomy approach. Patients with gastroparesis or obstructive tumors lying beyond the stomach commonly use jejunostomy feeding. Surgeons are reluctant to perform a gastrostomy on patients with esophageal cancer because they are concerned about future reconstructive surgery that uses the stomach as a conduit [3]. 

In 1990, O’Regan PJ and Scarrow GD pioneered the first laparoscopic feeding jejunostomy, providing the benefits of minimally invasive surgery. Since then, laparoscopic feeding jejunostomy has become a widely adopted method [4]. Previous studies have repeatedly shown that laparoscopic feeding jejunostomy is a safe treatment in comparison to open surgery. Nevertheless, it requires specific devices for insertion and fixation [3, 5–7]. 

A recent study found favorable results when utilizing the pure laparoscopic procedure, attributed to advancements in laparoscopic techniques [8]. Stam’s approach led to a high incidence of catheter site infections in the majority of the earlier laparoscopic reports [4, 6–8]. Recently, our institute has introduced laparoscopic feeding jejunostomy, which has become an increasingly popular option. Our surgical approaches differ from earlier studies by minimizing the use of commercial kit products. We expect this approach to reduce costs and enhance its implementation in surgical routines. Additionally, we have used Witzel’s tunnel technique, a popular method in open surgery. Our hypothesis was that the inclusion of a Witzel’s tunnel would result in a reduction in catheter site infections. Using the same approach in the open group allows for a more accurate comparison of results between the two groups.

The objective of this study was to compare the results of two distinct surgical approaches for feeding jejunostomy: open surgery and laparoscopic surgery. The focus was on assessing the perioperative outcomes, safety, and cost of each procedure.

## Patients and methods

### Population

All patients received feeding jejunostomy as an enteric route for enteric nutrition. The majority of these patients had stomach and esophageal cancer, but some had upper GI tract obstruction due to head and neck cancer (which made them ineligible for PEG), gastroparesis, or neurological patients with recurrent aspiration.

Research methodology: We conducted an amphispective cross-sectional study of patients who had feeding jejunostomy between January 2016 and June 2018 at Maharaj Nakorn Chiang Mai Hospital. We obtained the data retrospectively from the medical record database of Maharaj Nakorn Chiang Mai Hospital (Digicard 2007^®^) and prospectively from patients during their hospitalization for surgery. The first admission date, or the first consultation from another department, marked the start of data collection, which continued until the patients’ discharge from the hospital after their surgery. We categorized the patients into two distinct groups. Procedure options for feeding jejunostomy include open and laparoscopic techniques. We gathered data on the baseline characteristics of the participants, including their age, sex, BMI, ASA class, ECOG status, and co-morbidities. We also recorded details about the surgery, specifically the operational time and blood loss. Additionally, we assessed postoperative pain and documented any complications using the Clavien Dindo classification, distinguishing between those connected to the treatment and those unrelated. We also calculated the total admissions expenses. We initially recorded the data in paper format on a data collection form, then transferred it to a computer for later analysis.

### Exclusion criteria

We excluded patients who needed a feeding jejunostomy during the same operation but had not planned for it before surgery. If the patient underwent another surgical treatment during the same operation, we also excluded the data.

### Surgical technique

The following procedures were followed in a step-wise pattern.

Open Jejunostomy (OFJ) [9].

- We made an incision along the midline of the abdomen, specifically at the umbilicus area.
- Once inside the peritoneal cavity, we made an incision by puncturing the abdominal wall’s skin and fascia, slightly to the left of the rectus abdominus muscle.
- We used a clamp to firmly hold the jejunostomy tube’s tip and place it inside the peritoneal cavity.
- We identified Treitz’s ligament and selected a jejunal loop approximately 30 cm distal to it.
- We used a purse string suture of silk 3 − 0 on the antimesenteric side of jejunum.
- We performed an incision at the center of the purse string suture using electrocautery.
- We inserted the tip of the J-tube about 8 cm into the jejunum and then secured it with a purse string suture.
- We created Witzel’s tunnel by placing several interrupted 3 − 0 silk sutures at 1-cm intervals, 6 cm proximal to the enterotomy. We made sure to integrate the sutures into the bowel wall on both sides of the tube and bury the tube within the intestine’s wall.
- We used silk 3 − 0 sutures to secure the antimesenteric wall of the jejunum to the peritoneum.
- We tested the feeding’s functionality and looked for any leaks.
- We performed the abdominal closure in the usual manner.

**Fig. 1:**
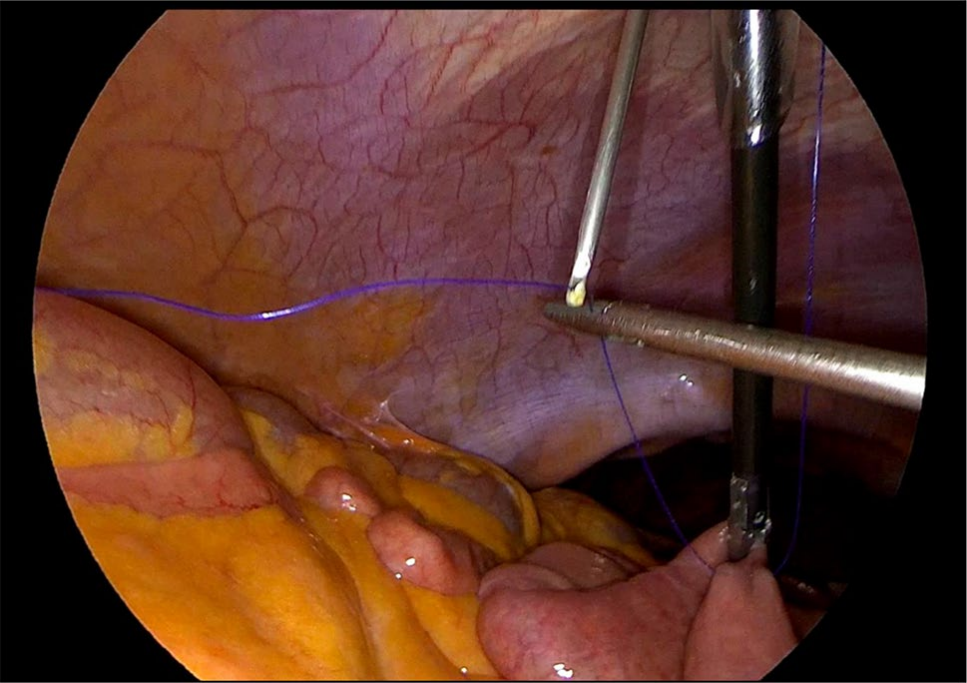
After the jejunostomy site was selected, the 1st cranial-side transfascial stitch was done

**Fig. 2:**
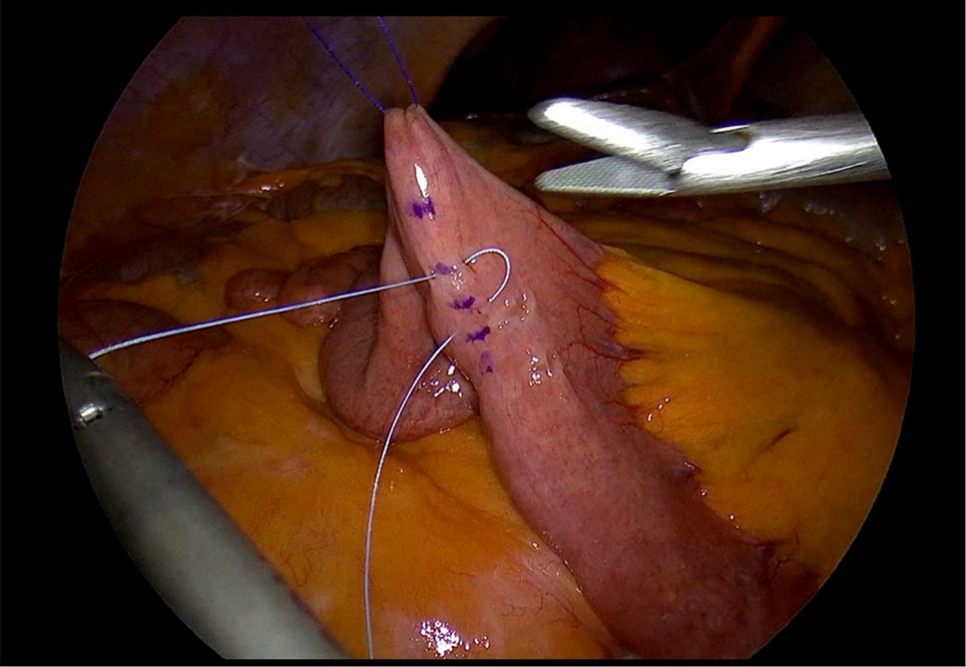
The purse string stitch was done at the planned enterotomy site but left untied

**Fig. 3:**
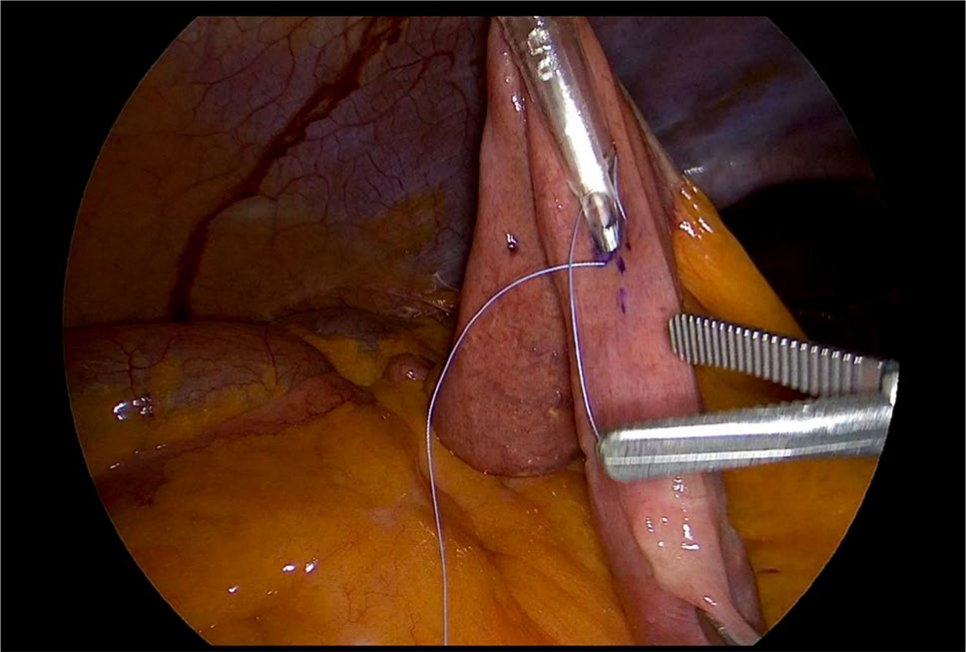
The tube was passed through the assistant trocar into the enterotomy using a trocar as a guide

**Fig. 4:**
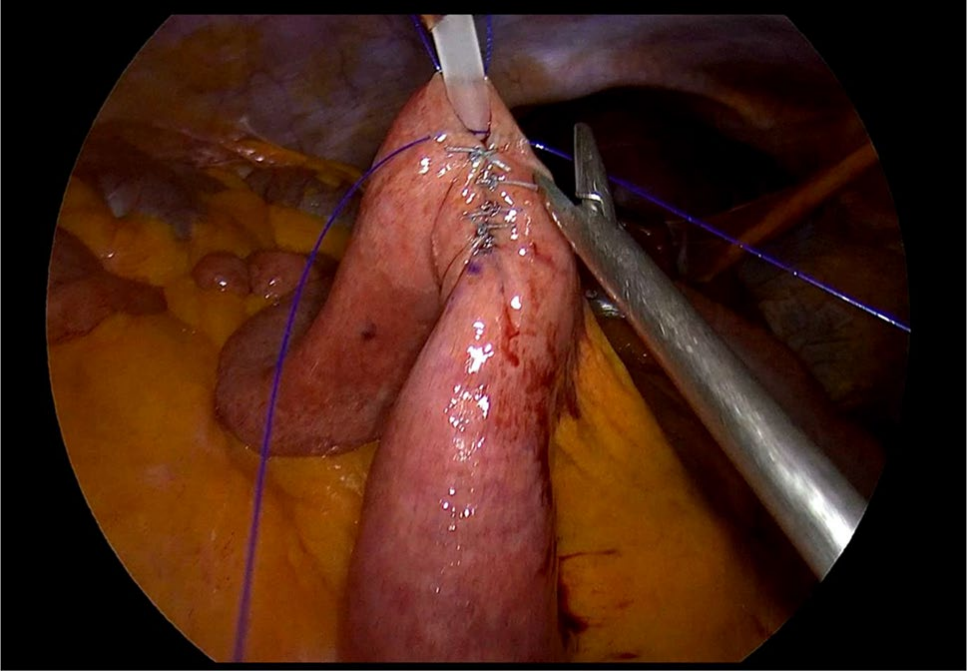
The Witzel’s tunnel was created from the enterotomy site to the planned 2nd trans-fascial stitch site

**Fig. 5:**
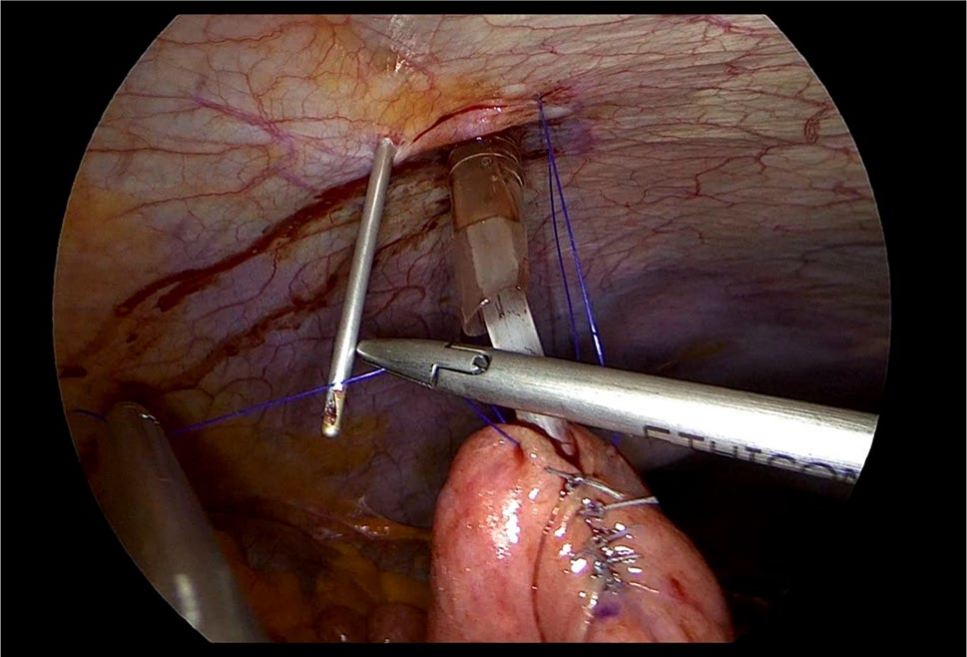
The 2nd trans-fascial stitch was done at the caudal side

Laparoscopic feeding jejunostomy (LFJ). (See Figs. 1, 2, 3, 4, and 5). 

- We positioned the 10 mm balloon camera port at the lower midline, approximately 10 cm below the umbilicus. We placed two working trocars in the left upper quadrant and left lower quadrant. We used a 12 mm trocar to facilitate the passage of the needle. We placed a 5 mm port at the right paraumbilical region, approximately 10 cm away from the umbilicus. This port not only served as an assistant trocar but also provided a passage for the feeding tube.
- We identified the ligament of Treitz and chose the loop consistent with the open approach.
- We used the Endo Close (Medtronic) for a first cranial-side abdominal transfacial stitch and Prolene 3/0 (Ethicon) for a hanging stitch.
- We used Ticron 3/0 (Medtronic) purse-string sutures with an intracorporeal suture technique and then performed an enterotomy using the same method as in an open procedure.
- A number 12 feeding tube was inserted via the right paraumbilical port as a jejunostomy tube.
- Using the trocar as a guide, we inserted the feeding tube 15 centimeters through the enterotomy.
- After achieving a satisfactory liquid flow, we secured the tube to the jejunum using a purse string suture.
- We used Ticron 3/0 (Medtronic) to create a 5-cm Witzel’s tunnel and Prolene 3/0 (Ethicon) to create a caudal-side transfacial suture.
- The surgeon removed the laparoscopic port and the operating instrument before closing the wound as usual.

### Statistical analysis

Continuous variables that were normally distributed were evaluated using a Student’s t-test. The non-normally distributed variables were evaluated using the Mann-Whitney U test. Fischer’s exact test was used to analyze categorical data. The multivariable analysis was conducted using logistic regression. Statistical analysis was conducted using STATA v.15 software.

## Results

This study included a total of seventy-four patients: thirty patients in the laparoscopic group and forty-four patients in the open surgery group. Table 1 provides a summary of the baseline characteristics. With the exception of the Preop ECOG performance status, all other gathered parameters were similar. The laparoscopic group had a higher percentage of patients with an ECOG score of zero, indicating better performance status.

**Table 1:** Baseline characteristics of patients

Parameter	*N* = 74 Laparoscopic (30)	Open (44)	*p*-value
Sex, n(%)			0.073
Male	25(83.33)	28(63.64)	
Female	5(16.67)	16(36.36)	
Age			
Mean(SD)	59.57(13.74)	56.61(14.57)	0.384
BMI, n(%)			0.907
<18	18(60.00)	25(56.82)	
18–25	11(36.67)	18(40.91)	
>25	1(3.33)	1(2.27)	
Mean (SD)	18.12(2.71)	17.90(13.17)	0.753
ASA class, n(%)			0.226
1	8(26.67)	5(11.36)	
2	20(66.67)	32(72.73)	
3	2(6.67)	6(13.64)	
4	0	1(2.27)	
Presence of Co-morbidity, n(%)	14(46.67)	27(61.36)	0.241
Indication for surgery, n(%)			0.486
1. Esophageal cancer	14(46.67)	24(54.55)	
2. Gastric cancer	9(30.00)	11(25.00)	
3. Other GI mechanical obstruction	5(16.67)	8(18.18)	
4. Gastroparesis	0	1(2.27)	
5. Reflux/Aspiration	2(6.67)	0	
Intention of surgery, n(%)			0.635
Pre-operative	11(36.67)	19(43.18)	
Palliative	19(63.33)	25(56.82)	
Previous abdominal surgery, n(%)	1(3.33)	2(4.55)	1.000
ECOG-pre-Op, n(%)			0.012
0	7(23.23)	3(6.82)	
1	12(40.00)	32(72.73)	
2	9(30.00)	4(9.09)	
3	1(3.33)	3(6.82)	
4	1(3.33)	2(4.55)	

The majority of patients who underwent feeding jejunostomy were male, with a mean age of 59.57 (SD = 13.74) and 56.61 (SD = 14.57) years in laparoscopic and open procedure groups, respectively. The mean BMI was 18.12 (SD = 2.71) and 17.90 (SD = 3.17) for laparoscopic and open procedures, respectively. A greater number of patients in the open group had co-morbidities; however, this difference was not statistically significant. The surgical indications included esophageal cancer, gastric cancer, and other upper GI mechanical obstructions. A small number of patients required a jejunostomy due to gastroparesis or aspiration.

The mean operative duration was 89.67 min (SD = 20.91) in the laparoscopic group and 91.84 min (SD = 22.79) in the open surgery group. There was a significantly different lower blood loss in the laparoscopic group during the procedure (9.87 ml, SD = 7.58) compared to the open group (24.32 ml, SD = 17.74) (*p* < 0.001). It is worth noting that none of the cases had any intraoperative complications.

The postoperative pain score in the first 24 h after patients returned from the recovery room to the ward was 4.28 (1.49) and 4.98 (1.69) (*p* = 0.076) in the laparoscopic and open groups, respectively. The mean morphine dosage was 3.3 mg (SD = 3.69) and 7.19 mg (SD = 4.00) (p = < 0.001) in the laparoscopic and open groups, respectively.

The initiation time for feeding did not differ significantly between the two groups (23 (IQR = 12.150) vs. 23 (IQR = 20), *p* = 0.785). However, the completion time of feeding tended to be earlier in the laparoscopic group (153 h. (IQR = 141) vs. 160 h. (IQR = 120), *p* = 0.969), although this difference was not statistically significant.

The length of hospital stay following laparoscopic surgery was marginally shorter in the laparoscopic group, with a median of 9.5 days (IQR = 9), compared to 10 days (IQR = 10.31) in the control group. However, this difference was not statistically significant (*p* = 0.574). The cost of laparoscopic feeding jejunostomy is significantly higher (median 16,410 Thai Baht, IQR = 5140) compared to conventional feeding jejunostomy (median 11,685 Thai Baht, IQR = 5375), with a p-value of less than 0.001. However, the overall cost tends to be lower in the laparoscopic group (median 105,147 Thai Baht, IQR = 179,744) compared to the conventional group (median 116,198 Thai Baht, IQR = 108,276), but this difference is not statistically significant (*p* = 0.387) (See Table 2).

**Table 2:** Comparison of operative outcomes

Parameter	Laparoscopic	Open	*p*-value
Operative time(min)			
Mean (SD)	89.67(20.91)	91.84(22.79)	0.678
EBL (ml)			
Mean (SD)	9.87(7.58)	24.32(17.74)	< 0.001
Median (IQR)	5(15)	20(40)	< 0.001
Total wound length			
Mean (SD)	3.05(0.11)	10(1.89)	< 0.001
Incidence of Intra-op complications, n(%)	0	0	NA
Post op pain score in 1st 24 h			
Mean (SD)	4.28(1.49)	4.98(1.69)	0.076
Total morphine consumption (mg)			
Mean (SD)	3.3(3.69)	7.19(4.00)	< 0.001
Feeding initiated (hr.)			
Median (IQR)	23(12.5)	23(20)	0.785
Feeding accomplished (hr.)			
Median (IQR)	153 (141)	160(120)	0.969
Post op hospital stay (day)			
Median (IQR)	9.5 (9)	10 (10.31)	0.574
Dead, n(%)	1(3.33)	3(6.82)	0.642
Operative Costs (THB)			
Median (IQR)	16,410 (5,140)	11,685(5,375)	< 0.001
Total Costs (THB)			
Median (IQR)	105,147(179,744)	116,198(108,276)	0.387

The incidence of postoperative complications is slightly lower in the LFJ group, although this difference is not statistically significant. The LFJ group experienced Grade I and II complications, including one feeding symptom, one instance of bleeding at the tube site, and one tube blockage. In contrast, the OFJ group experienced three eating-related symptoms, two cases of upper gastrointestinal hemorrhage, and two tube obstructions. This study documented four cases of reoperation. Following surgery, two individuals in the LFJ group experienced small bowel blockage due to the kinking of the jejunum at the trans-fascial stitch site. Laparoscopic techniques successfully addressed these obstructions by removing the transfascial suture and realigning the jejunum. Three additional surgical procedures were required in the OFJ group due to tube misplacement, jejunum blockage, and mesenteric ischemia. We removed the misplaced tube and re-inserted it into the correct position. A jejuno-jejunostomy bypass procedure successfully resolved the obstruction. However, the patient’s ischemic bowel situation was severe; the patient passed away shortly after the surgery. A single individual in the LFJ group died from hospital-acquired pneumonia. Three individuals in the OFJ group died from hospital-acquired pneumonia, a thyroid storm, and intestinal ischemia. The multivariate study of complications indicates that no significant factor influenced the outcome (See Table 3).

**Table 3:** Complications according to clavien-dindo classification between laparoscopic FJ and open FJ

Parameter	Lap FJ	Open FJ	*P*-Value
Clavien-Dindo grade, n (%)			0.846
0	24(80)	33(75.0)	
1–2	3(10)	7(15.91)	
3–4	3(10)	4(9.09)	

## Discussion

Enteric nutritional treatment is a crucial part of therapy for upper gastrointestinal cancer due to the high occurrence of malnutrition resulting from tumor obstructive symptoms [1]. The majority of our research sample also had a significantly low body mass index (BMI) of less than 18. Malnutrition not only hinders patients from receiving therapy but also affects the outcome. The tumor’s response rate to chemoradiation is dependent on the patient’s nutritional status, which has a direct impact on the prognosis and overall treatment outcome [10]. Jejunostomy feeding is a vital surgical procedure that allows for enteral nourishment, which is essential for patients with upper gastrointestinal cancer. So far, laparoscopic feeding jejunostomy has several advantages in terms of less invasive surgical procedures. Consequently, this treatment is gaining popularity at an escalating rate [11]. 

Prior studies into the application of laparoscopic techniques frequently require the employment of specialized instruments and adjustments to the traditional techniques. The jejunostomy insertion kit is commonly used to place a tube into the intestines [3, 5, 6, 12, 13]. Our method involves the use of a laparoscopic trocar as a guide to safe insertion of the jejunostomy tube into the distal side of the jejunum. This technique allows for precise control of the insertion direction and eliminates the need for a specialized kit. Various fixing methods have been documented. Duh QY et al. proposed the utilization of T fasteners, a specialized fixation device, to secure the intestine to the abdominal wall [5]. In our investigation, we employed the transfacial fixation technique, which involves the use of a fascial closure needle. It provides a robust link between the gut and the abdominal wall. The instrument is inexpensive and readily accessible. The suture also offered good surgical exposure and facilitated tube insertion by providing opposing force. This strategy enabled the integration of the minimally invasive technique into regular surgical procedures by reducing the need for specialized instruments. In our investigation, we also utilized Witzel’s tube technique, which bears resemblance to the open surgery procedure. In contrast, the laparoscopic feeding jejunostomy approach commonly employs Stamm’s technique because of its simplicity [3, 14, 15]. The occurrence of catheter site infection/irritation was reported in various studies, the incidence ranging from 0.7 to 29.1% [6, 14–16]. Mastoridis et al. conducted a comparative study comparing laparoscopic and open techniques. They showed that there was no significant difference between the infection rate associated with laparoscopic Stam’s technique and open short (2 cm) Witzel’s approach (12.5% vs. 7.9%, *p* = 0.99) [6]. Young et al. modified the surgical approach from Stamm’s to Witzel’s in response to the severe catheter site infection. This change resulted in a reduction of the infection rate from 1.8 to 0% [16]. A recent publication by P Varshney et al., which also investigated the outcomes of using Witzel’s approach, reported a concordant result indicating no occurrence of surgical site infection in the study [17]. We performed Witzel’s tunnel procedure with a length of 5 cm. There were no reports of surgical site infection in our study.

In general, laparoscopic procedures typically are of longer duration compared to the same treatment conducted with an open approach [18]. Several publications have found a longer duration for laparoscopic feeding jejunostomy compared to the open procedure, particularly when using a purely laparoscopic approach (159–180 min) [8, 17]. In our study, the mean operating time was marginally shorter in LFJ compared to the open technique, although this difference was not statistically significant (89.67 vs. 91.84 min). From our perspective, laparoscopic feeding jejunostomy offers several benefits. These include the ease of identifying the ligament of Treitz, which is a critical step in selecting the enterotomy bowel segment; stitching the enterotomy loop to the abdominal wall becomes easier; and eliminating the need to close the long laparotomy wound.

In our study, the decreased total wound length (3.05 cm vs. 10 cm, *p* < 0.001) led to a reduced amount of blood loss and lower morphine use. The postoperative pain score was lower in the LFJ group, but there was no statistically significant difference between the two groups. Nevertheless, the LFJ group exhibited a statistically significant and clinically meaningful reduction in morphine intake, with barely half the amount of morphine used compared to the control group (3.3 vs. 7.19, *p* < 0.001). The outcomes of our study were consistent with recent research that used a minimally invasive approach [3, 4, 8]. 

There was no difference in the time at which postoperative feeding was started between the two groups. According to our institute’s policy, we would begin feeding the patient on the day following the surgery, provided that their condition remained stable. The duration of feeding was marginally shorter in the LFJ group; however, this difference was not statistically significant. In the LFJ group, the duration of stay was shorter; however, there was no statistically significant difference in the time. The results of the study were similar to those of a previous study that compared open and laparoscopic procedures and found that there was no significant difference in the number of complications related to feeding (11.1% vs. 12.5%, *p* = 0.99 for open and laparoscopic procedures, respectively) [6]. 

The LFJ group incurred significantly greater operative cost due to the inclusion of an additional laparoscopic device. Interestingly, the median total expense for admission was not significantly lower in the laparoscopic group, despite the fact that the operating cost was greater. As a result, the minimally invasive procedure did not increase the overall cost of therapy.

The findings of the study indicated that there were no statistically significant differences in complication outcomes between the two groups. In our study, eating intolerance accounted for the majority of grade 1–2 complications, contrary to previous research reports that focused on wound infections [11]. This result may have been attributable to our implementation of Witzel’s tunnel technique. The incidence of grade 3–4 complications in our study was approximately 10% in both groups, which was consistent with the findings of prior studies that reported a range of 9.5–25% [12, 17]. Overall, we deemed both surgical procedures safe, with a relatively low occurrence of intraoperative and postoperative complications. The primary reason for reoperation in our investigation was the occurrence of jejunum kinking at the fixation site, which was most prevalent during the initial phase of the trial (first ten cases). However, after adjusting the fixation alignment from lateromedial to craniocaudal, there were no further instances of this complication.

There are limitations to this study. The study design employed was a retrospective cohort study; therefore, the possibility of the impact of confounding factors could not be ruled out. Frequently, the inclusion of many supplementary treatments during the same hospital admission led to an extended duration of stay and increased overall expenses. Additional research conducted using a randomized controlled trial design will provide valuable insights into the efficacy of the laparoscopic feeding jejunostomy technique.

## Conclusion

The laparoscopic feeding jejunostomy demonstrated safety and decreased postoperative morphine consumption. The overall expense in comparison to the open approach remains unchanged. The Witzel’s tunnel technique may reduce the risk of infection at the jejunostomy site.

## Data Availability

Data is provided as supplementary information files.

## Abbreviations

ASA: American Society of Anesthesiologists
BMI: body mass index
ECOG: Eastern Cooperative Oncology Group
GI: gastrointestinal
IQR: interquartile range
J-tube: jejunostomy tube
LFJ: laparoscopic feeding jejunostomy
OFJ: open feeding jejunostomy
PEG: percutaneous endoscopic gastrostomy
SD: standard deviation
THB: Thai baht
